# Kidney outcomes of coenzyme Q10 supplementation in patients with genetically confirmed CoQ10 nephropathy in Japan

**DOI:** 10.1007/s10157-026-02917-7

**Published:** 2026-07-11

**Authors:** China Nagano, Katsumi Ushijima, Yuko Tezuka, Takayuki Okamoto, Yasuhiro Inaba, Akinori Miyazono, Naoko Takeda, Taro Aoki, Akira Mizutani, Hiroki Miyano, Masaki Yamamoto, Mariko Imai, Takehiko Kawaguchi, Toshiyuki Komiya, Masayuki Ishihara, Yoshiki Nagao, Naoaki Mikami, Yuta Inoki, Nana Sakakibara, Tomoko Horinouchi, Tomohiko Yamamura, Shingo Ishimori, Kandai Nozu

**Affiliations:** 1 Department of Pediatrics, Kobe University Graduate School of Medicine, 7-5-1 Kusunoki-Cho, Chuo-Ku, Kobe, Hyogo 650-0017 Japan; 2 Department of Pediatrics, Yokkaichi Municipal Hospital, Yokkaichi, Japan; 3 Department of Pediatrics, Ehime Prefectural Imabari Hospital, Imabari, Japan; 4 Department of Pediatrics, Hokkaido University Hospital, Sapporo, Japan; 5 Department of Pediatrics, Kagoshima University Graduate School of Medical and Dental Sciences, Kagoshima, Japan; 6 Department of Medicine, Shiga University of Medical Science, Shiga, Japan; 7 Internal Medicine 1, Hamamatsu University School of Medicine, Hamamatsu, Japan; 8 Department of Pediatrics, Dokkyo Medical University Saitama Medical Center, Saitama, Japan; 9 Department of Pediatrics, Juntendo University Nerima Hospital, Tokyo, Japan; 10 Department of Pediatric Nephrology, Seirei Hamamatsu General Hospital, Hamamatsu, Japan; 11 Department of Nephrology, National Hospital Organization Chiba Medical Center Chibahigashi National Hospital, Chiba, Japan; 12 Department of Nephrology, Japanese Red Cross Otsu Hospital, Otsu, Shiga Japan; 13 Department of Pediatrics, Kochi Medical School, Kochi University, Kochi, Japan; 14 Department of Nephrology and Rheumatology, Tokyo Metropolitan Children’s Medical Center, Tokyo, Japan

**Keywords:** CoQ10 nephropathy, COQ6, COQ8B, CoQ10 supplementation

## Abstract

**Background:**

Coenzyme Q10 (CoQ10) nephropathy is a rare mitochondrial kidney disease caused by defects in CoQ10 biosynthesis and represents a unique form of steroid-resistant nephrotic syndrome with a disease-specific therapy. However, data on treatment outcomes of this disease in Japanese patients remain limited.

**Methods:**

We conducted a retrospective observational study of Japanese patients. Patients with a genetically confirmed diagnosis of CoQ10 nephropathy who received CoQ10 supplementation and had available longitudinal clinical data were included. Changes in the urinary protein-to-creatinine ratio (UPCR) and estimated glomerular filtration rate before and after treatment were evaluated, and adverse events were assessed.

**Results:**

Twelve patients were included in the analysis. The median age at treatment initiation was 9.0 years, and *COQ8B* was the predominant causative gene (n = 11). One patient harbored a *COQ6* variant. CoQ10 supplementation was initiated at a median dose of 10.0 mg/kg/day. The median UPCR decreased from 1.66 g/gCr at baseline to 0.19 g/gCr at 12 months, and 6/7 (86%) patients with available 12-month data achieved a ≥ 50% reduction in proteinuria. Kidney function remained stable, and no patients progressed to end-stage kidney disease during a median follow-up of 28.8 months. Adverse events were mild and did not lead to treatment discontinuation.

**Conclusions:**

In Japanese patients with CoQ10 nephropathy, CoQ10 supplementation was associated with a substantial reduction in proteinuria and stabilization of kidney function. These findings indicate the importance of early genetic diagnosis and prompt initiation of targeted therapy for this treatable hereditary kidney disease.

**Supplementary Information:**

The online version contains supplementary material available at 10.1007/s10157-026-02917-7.

## Introduction

Coenzyme Q10 (CoQ10) nephropathy is a form of mitochondrial disease caused by primary deficiency of CoQ10, which is an essential lipid-soluble electron carrier in the mitochondrial respiratory chain. CoQ10 shuttles electrons from complexes I and II to complex III, enabling oxidative phosphorylation and ATP production, while also functioning as a potent antioxidant that protects cell membranes from oxidative damage [1, 2]. Mitochondria-rich tissues, such as the kidney, heart, and brain, are particularly susceptible to CoQ10 depletion. In the kidney, podocytes are highly dependent on mitochondrial energy metabolism, and their injury in CoQ10 deficiency manifests as proteinuria, focal segmental glomerulosclerosis, and often steroid-resistant nephrotic syndrome [3, 4].

The molecular basis of CoQ10 nephropathy lies in pathogenic variants affecting the multi-step biosynthetic pathway of CoQ10. To date, variants in more than 10 genes, including *PDSS1*, *PDSS2*, *COQ2*, *COQ4*, *COQ5*, *COQ6*, *COQ7*, *COQ8A*, *COQ8B*, and *COQ9*, have been implicated [5, 6]. Among these, *COQ2*, *COQ6*, and *COQ8B* are most commonly associated with isolated or predominant kidney disease. While most monogenic forms of steroid-resistant nephrotic syndrome have no disease-specific therapy, CoQ10 nephropathy is exceptional in that oral supplementation with high doses of CoQ10 can restore mitochondrial function to some extent, reduce proteinuria, and slow progression to end-stage kidney disease [3, 7, 8]. Importantly, treatment efficacy is strongly time-dependent in that benefits are greatest when supplementation is initiated before irreversible glomerular sclerosis has occurred.

Epidemiological observations suggest regional variation in the prevalence and genetic architecture of CoQ10 biosynthesis disorders. A recent comprehensive review of primary CoQ10 deficiency summarized its broad clinical spectrum, ranging from isolated nephropathy to multisystem disease, and highlighted differences in reported causative genes across populations [7]. An example of these differences is that a recurrent *COQ4* variant has been reported in southern Chinese patients, and variants in *COQ6* and *COQ8B* appear relatively more frequent in East Asian cohorts than in Western populations [5, 9, 10]. These findings suggest possible population-specific variant spectra that may affect the diagnosis and management strategies.

In Japan, unique nationwide screening programs, including the health checkup at 3 years of age and the school-based urinary screening system, allow for earlier detection of glomerular diseases than in many other countries. This healthcare framework enables a prompt diagnosis and early initiation of treatment for pediatric kidney diseases, representing a major strength of the Japanese medical system [11, 12]. In this context, establishing evidence of CoQ10 nephropathy in Japanese patients is particularly important because early recognition of this treatable condition through genetic testing could further improve clinical outcomes. Despite these observations, comprehensive data on the prevalence, genetic spectrum, clinical presentation, and treatment response of CoQ10 nephropathy in Japanese patients remain limited. Most published Japanese cases are isolated reports or small series [13], which lack systematic analysis of genotype–phenotype correlations and longitudinal outcomes. This gap in knowledge may delay recognition of a treatable cause of steroid-resistant nephrotic syndrome and lead to missed opportunities for timely intervention. Identifying affected individuals early through genetic testing has important implications for the prognosis because of the availability of an effective, low-risk therapy.

The present study aimed to define the clinical and genetic landscape of CoQ10 nephropathy in the Japanese population, characterize treatment responses to CoQ10 supplementation, and highlight the critical importance of early diagnosis in optimizing kidney outcomes for this rare but treatable mitochondrial kidney disease.

## Materials and methods

### Study participants

After obtaining informed consent, clinical data and blood samples were collected from individuals with proteinuria in whom hereditary kidney disease was clinically suspected across Japan. Genetic testing was performed at Kobe University Graduate School of Medicine using a targeted sequencing panel for hereditary kidney diseases. The study was approved by the Institutional Review Board of Kobe University Graduate School of Medicine (IRB approval number: 301).

Between January 2016 and August 2025, patients with proteinuria and suspected hereditary kidney disease were referred for genetic testing. Among these, 16 patients were genetically diagnosed with coenzyme Q10 (CoQ10)-related nephropathy. Longitudinal clinical data were retrospectively requested from the referring institutions, and complete clinical data were obtained for 14 patients. Of these, 12 patients who received coenzyme Q10 supplementation and had available follow-up data after treatment initiation were included in the final retrospective analysis (Table 1). One of these patients (Case 3) had been partially reported previously [13], with a description of the clinical course up to an earlier time point.

**Table 1:** Genetic characteristics and treatment status of patients with COQ10 nephropathy

No.	ID	Gene	Sex	Age at diagnosis (y)	Zygosity	Variant	COQ10 treatment
1	Neph36	*COQ8B*	F	8	homozygous	c.737G > A, p.(Ser246Asn)	Yes
2	Neph56	*COQ8B*	F	4	compound heterozygous	c.532 C > T, p.(Arg178Trp) c.737G > A, p.(Ser246Asn)	Yes
3	Neph97	*COQ6*	M	0.9	compound heterozygous	c.782C > T, p.(Pro261Leu) Large Deletion	Yes
4	Neph160	*COQ8B*	M	5	compound heterozygous	c.532 C > T, p.(Arg178Trp) c.737G > A, p.(Ser246Asn	Yes
5	Neph250	*COQ8B*	F	44	homozygous	c.737G > A, p.(Ser246Asn)	No
6	Neph364	*COQ8B*	M	28	homozygous	c.737G > A, p.(Ser246Asn)	Yes
7	Neph501	*COQ8B*	F	9	homozygous	c.737G > A, p.(Ser246Asn)	Yes
8	Neph652	*COQ8B*	M	6	homozygous	c.737G > A, p.(Ser246Asn)	Yes
9	Neph653	*COQ8B*	F	9	compound heterozygous	c.646delG, p.(Ala216Profs*) c.737G > A, p.(Ser246Asn)	Yes
10	Neph814	*COQ8B*	F	28	homozygous	c.737G > A, p.(Ser246Asn)	Yes
11	Neph859	*COQ8B*	M	43	homozygous	c.737G > A, p.(Ser246Asn)	Yes
12	Neph868	*COQ8B*	M	17	homozygous	c.737G > A, p.(Ser246Asn)	Yes
13	Neph960	*COQ8B*	M	3	homozygous	c.737G > A, p.(Ser246Asn)	Yes
14	Neph986	*COQ8B*	F	5	homozygous	c.737G > A, p.(Ser246Asn)	No

Variants are described according to the following reference transcripts: COQ8B: NM_024876.3; COQ6: NM_0182476.2

### Genetic analysis

Genomic DNA was extracted from peripheral blood using the Quick Gene Mini 80 system or Quick Gene Auto 12S (Wako Pure Chemical Industries, Ltd., Tokyo, Japan) and used for targeted sequencing and Sanger sequencing. Targeted sequencing using next-generation sequencing was conducted for genes responsible for inherited kidney diseases, which included genes involved in coenzyme Q10 biosynthesis. The sample library for next-generation sequencing was prepared using the HaloPlex or SureSelect XT2 custom capture library 0.5–2.9 Mb (Agilent Technologies, Santa Clara, CA, USA), in accordance with the manufacturer’s workflow. All indexed DNA samples were amplified by polymerase chain reaction and sequenced using the MiSeq platform (Illumina, San Diego, CA, USA). In one patient with a *COQ6* variant, a pathogenic variant on one allele had been identified by a custom array-based analysis before this study, as previously described [13].

### Data collection

Participating physicians at referring hospitals were requested to provide clinical follow-up information using a standardized Excel form. Data collected included baseline characteristics, such as age, sex, age at diagnosis, causative gene, and variant classification. Disease onset was defined as the age at first detection of proteinuria. The interval from disease onset to initiation of CoQ10 supplementation was calculated for each patient. Pre-treatment laboratory values of the urine protein-to-creatinine ratio (UPCR, g/gCr), serum creatinine concentrations (mg/dL), estimated glomerular filtration rate (eGFR, mL/min/1.73 m^2^), height, and weight were collected. Furthermore, data of treatment details, including the date of CoQ10 initiation, formulation, dose (mg/kg/day), dosing frequency, and concomitant medications (especially renin–angiotensin system inhibitors), were collected. Follow-up measurements included the UPCR, serum creatinine concentrations, the eGFR, height, weight, and any adverse events at each follow-up. The data of adverse events comprised the type, severity, and relation to CoQ10 therapy. Longitudinal dosing data were collected at each follow-up visit, and summary statistics were calculated at both the visit level and patient level.

### Statistical analysis

Continuous variables are shown as the median and interquartile range. Comparisons between baseline and post-treatment values were performed using the Wilcoxon signed-rank test, as appropriate. A two-sided *p* value < 0.05 was considered statistically significant. All statistical analyses were performed using R software (version 4.4.3).

## Results

Between January 2016 and August 2025, 997 patients with proteinuria and suspected hereditary kidney disease underwent genetic testing. Among them, 16 patients were diagnosed with CoQ10-related nephropathy. Longitudinal clinical data were available for 14 patients, and 12 patients who received coenzyme Q10 supplementation were included in the final retrospective analysis (Table 1). The median post-treatment follow-up duration was 28.8 months (7.3–53.5 months).

### Baseline characteristics

At baseline (pre-treatment), the median age was 9.0 years (5.8–20.5) and there were eight (66.7%) male patients. The causative genes included *COQ6* (n = 1) and *COQ8B* (n = 11). The median UPCR was 1.66 g/gCr (1.25–3.39), and the median eGFR was 80.9 mL/min/1.73 m^2^ (70.1–95.7 mL/min/1.73 m^2^).

The age at initiation of CoQ10 supplementation was a median of 9.7 years (5.0–23.0) in the overall cohort. Among pediatric patients, the median age at treatment initiation was 7.4 years (4.6–9.8). Disease onset was defined as the first detection of proteinuria. The median interval from disease onset to initiation of CoQ10 supplementation was 32.4 months (14.3–94.7) in the overall cohort (range 4.7–242.7; Supplementary Table S1). This interval was shorter in pediatric patients (median 18.8 months, IQR 7.6–36.6) than in adult patients (median 113.9 months, IQR 101.1–178.3).

During longitudinal follow-up, CoQ10 doses were adjusted according to clinical response and tolerability. Across all patients, the median CoQ10 dose at treatment initiation was 10.0 mg/kg/day (7.5–12.1), while the median of individual median doses during follow-up was 13.1 mg/kg/day. The median of individual maximum doses was 21.5 mg/kg/day (range, 7.9–30.0 mg/kg/day), and the overall observed dose range across all visits was 0.4–30.0 mg/kg/day. Dose escalation to ≥ 20 mg/kg/day was performed in several patients, reflecting individualized dose optimization in real-world clinical practice.

### Treatment exposure

Coenzyme Q10 supplementation was initiated at a median dose of 10.0 mg/kg/day (7.5–12.1 mg/kg/day), administered twice daily. Ten (83.3%) patients received concomitant renin–angiotensin system inhibitors.

### Primary outcome: proteinuria

**Fig. 1:**
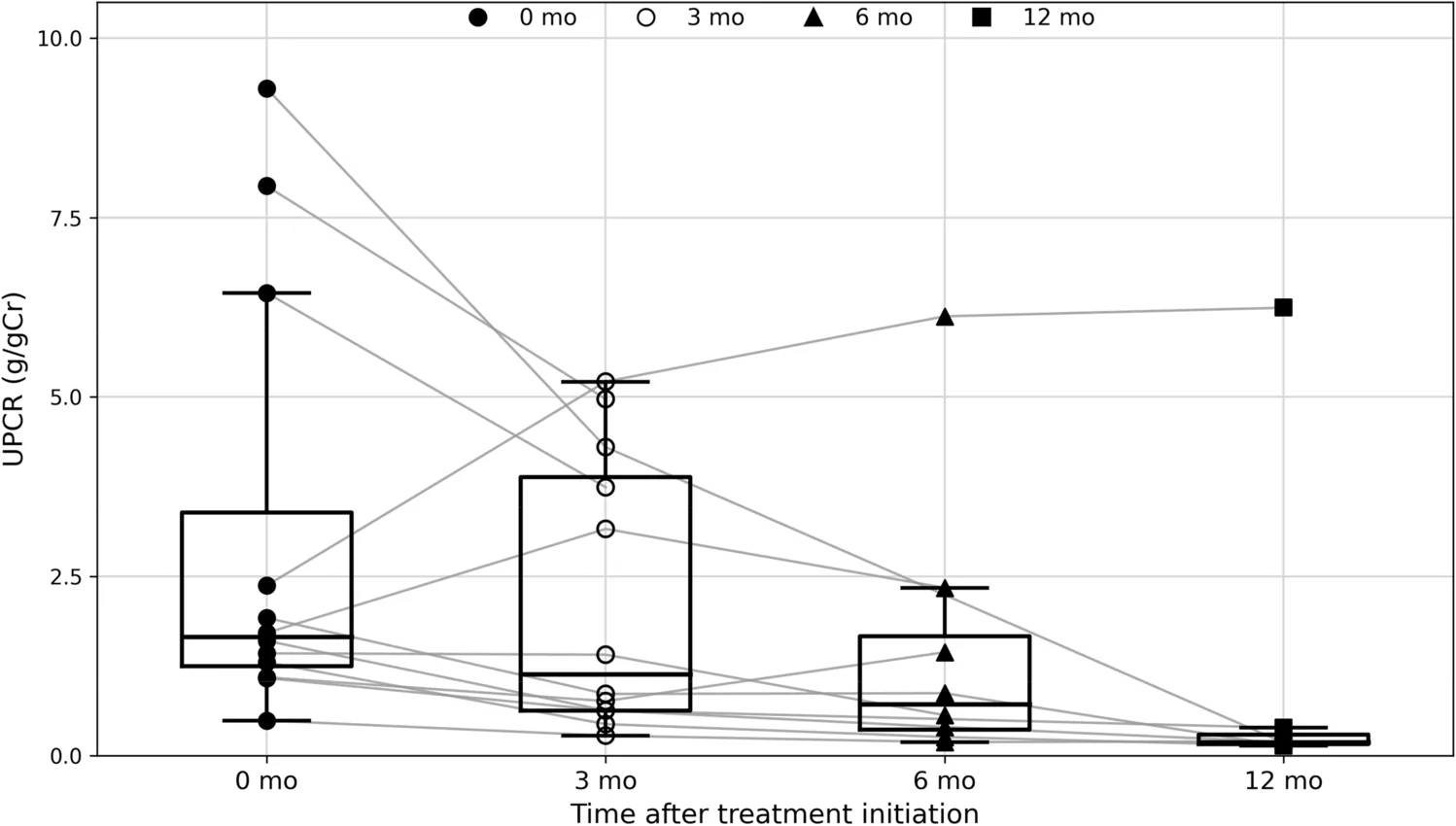
Changes in proteinuria over time after initiation of coenzyme Q10 supplementation. Boxplots indicate the median and interquartile range, with individual patient values overlaid. Thin lines connect paired measurements from the same patient

The median UPCR decreased from 1.66 g/gCr at baseline to 1.13 g/gCr, 0.72 g/gCr, and 0.19 g/gCr at 3, 6, and 12 months after treatment initiation, respectively. Paired comparisons using the Wilcoxon signed-rank test did not reach significance at any time point (Table 2, Fig. 1). Among patients with available 12-month UPCR data, 6 of 7 (86%) patients achieved a ≥ 50% reduction in the UPCR compared with baseline. One patient did not show a reduction in proteinuria following the initiation of coenzyme Q10 supplementation and experienced persistent gastrointestinal symptoms, including diarrhea, during the treatment period.

**Table 2:** Changes in the UPCR over 12 months after initiation of coenzyme Q10 supplementation

Timepoint	UPCR median (IQR), g/gCr	Δ from baseline	*p*-value (paired)	n measured
Baseline	1.66 (1.25–3.39)	–	–	12
3 months	1.13 (0.63–3.88)	− 0.52	0.11	12
6 months	0.72 (0.37–1.67)	− 0.65	0.55	8
12 months	0.19 (0.16–0.30)	− 1.41	0.22	7

UPCR, urinary protein-to-creatinine ratio; IQR, interquartile range

### Secondary outcomes: kidney function

Among patients with available paired eGFR data at 12 months (n = 6), the median eGFR increased from 93.9 mL/min/1.73 m^2^ at baseline to 106.6 mL/min/1.73 m^2^ at 12 months (median Δ: + 9.0 mL/min/1.73 m^2^), but this change was not significant (*p* = 0.69). The median annualized eGFR slope was − 1.7 mL/min/1.73 m^2^/year (95% confidence interval, − 4.9 to 3.1).

### Long-term outcomes

Over a median post-treatment follow-up of 28.8 months (maximum: > 90 months), a reduction in proteinuria was sustained in most patients (Supplemental Fig. S1). No patients reached the composite kidney outcome of a ≥ 40% eGFR decline, end-stage kidney disease, or kidney replacement therapy (Supplemental Fig. S2). An age-based longitudinal plot further illustrated the trajectories of kidney function relative to patient age and timing of treatment initiation (Supplemental Fig. S3).

### Subgroup analysis by age

**Fig. 2:**
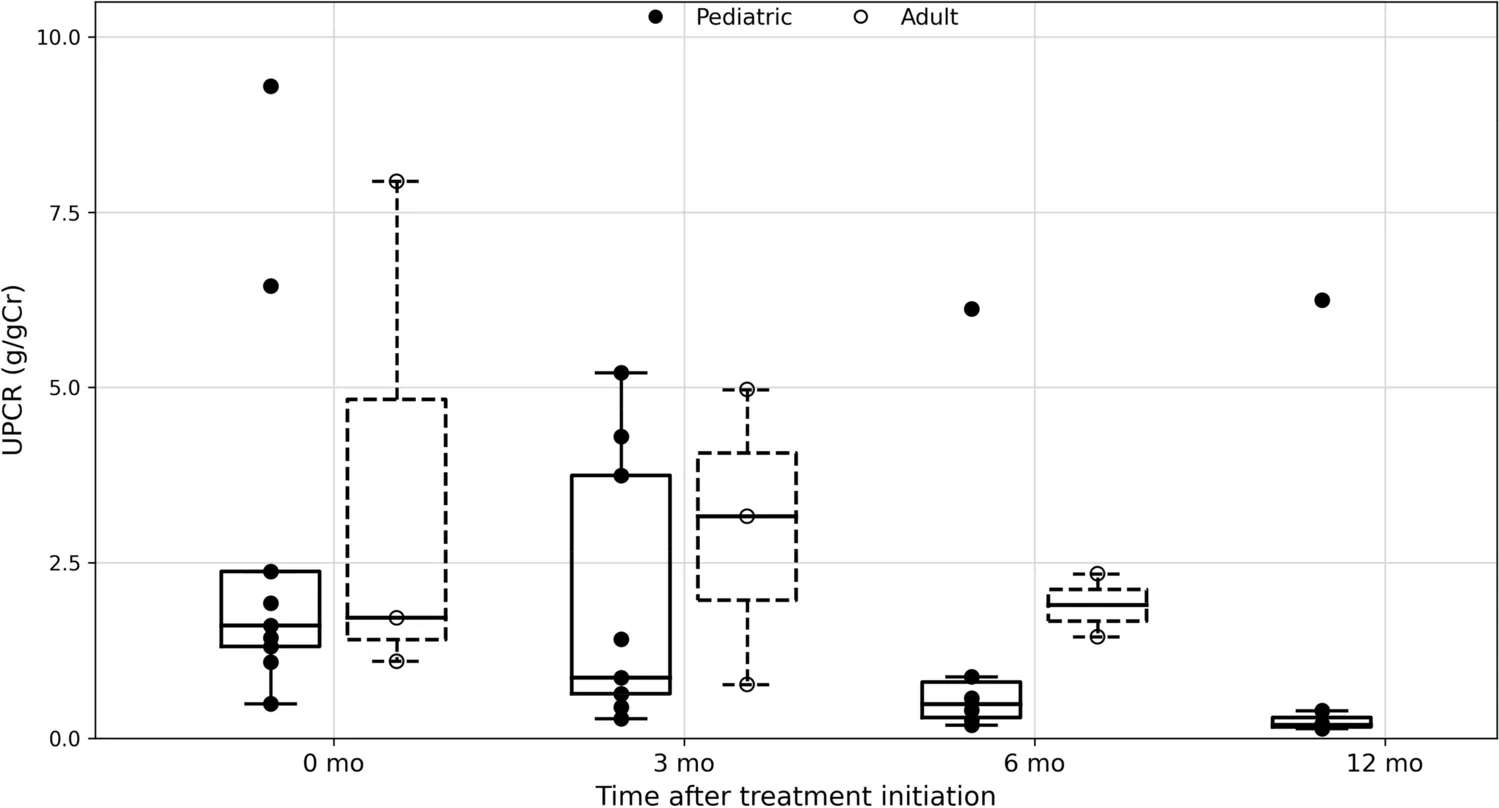
Changes in the UPCR over time stratified by age group. Box plots represent the distribution of UPCR values in pediatric and adult patients at each time point, with individual data points overlaid. Adult patients are indicated by open symbols. UPCR values were summarized using representative measurements within ± 2 months of each predefined time point

When the patients were stratified by age group, with three adult patients and the remaining patients classified as pediatric cases, no significant differences in the UPCR were observed between adult and pediatric patients at baseline or at any follow-up time point (Fig. 2). Although both groups showed a general trend of a reduction in proteinuria following coenzyme Q10 supplementation, the magnitude of change did not differ appreciably between adult and pediatric patients at 3 or 6 months. A statistical comparison at these time points was not performed owing to the limited number of adult patients with available 12-month data.

### Safety

Adverse events were reported in three (25.0%) patients. Gastrointestinal symptoms, including diarrhea, gastric discomfort, and vomiting, were observed in two (16.7%) patients, and vomiting occurred at treatment initiation in one patient. Neurological symptoms, such as dizziness or somnolence, were observed in two (16.7%) patients, although the causal relationship with coenzyme Q10 supplementation was uncertain in one case. No cases of skin rash or elevated transaminases were reported. Importantly, no treatment-related serious adverse events or treatment discontinuation occurred during the follow-up period.

## Discussion

In this study of a nationwide cohort of patients with genetically confirmed CoQ10 nephropathy, we observed that CoQ10 supplementation was associated with a substantial reduction in proteinuria and stabilization of kidney function over a 12-month follow-up. The median UPCR was decreased by approximately 90% at 12 months compared with baseline, and the eGFR remained largely stable. Adverse events were infrequent and mild, and consisted mainly of gastrointestinal discomfort, with no treatment-related serious adverse events. These findings are particularly notable because of the progressive nature of CoQ10 nephropathy and the lack of alternative disease-modifying therapies.

Despite these favorable short-term outcomes, heterogeneity in long-term disease trajectories was observed. During the extended follow-up, one pediatric patient showed a gradual increase in proteinuria and decline in kidney function after an initial response to coenzyme Q10 supplementation. This clinical deterioration coincided with documented periods of reduced adherence to therapy. By contrast, another patient did not show a reduction in proteinuria and experienced persistent gastrointestinal symptoms, including diarrhea, during the treatment period, which may have affected drug absorption. Maintaining long-term adherence, particularly in pediatric patients, may be critical for sustaining the therapeutic benefit because of the chronic nature of coenzyme Q10 nephropathy and the requirement for lifelong supplementation. In addition, factors affecting gastrointestinal absorption may affect the treatment response. However, because adherence and drug bioavailability were not systematically quantified and the number of non-responders was small, other factors, such as disease severity and intercurrent clinical events, may also have contributed. Our results align with previous reports suggesting that early initiation of CoQ10 supplementation in primary CoQ10 deficiencies, particularly COQ8B- and COQ6-related nephropathy, can attenuate proteinuria and slow the decline in kidney function [1–3, 5, 14–16]. An early genetic diagnosis and initiation of CoQ10 supplementation in COQ8B nephropathy is associated with slower progression of kidney dysfunction and a reduced risk of early end-stage kidney disease compared with delayed diagnosis and treatment [17]. Additionally, Stańczyk et al. [18] reported marked improvement and sustained remission of proteinuria in a child with COQ6-related nephropathy following CoQ10 therapy. In Japan, nationwide pediatric screening programs, including the health checkup at 3 years of age and the school-based urinary screening system, facilitate early detection of asymptomatic urinary abnormalities. These systems provide an opportunity for earlier clinical evaluation and genetic testing, potentially leading to an earlier diagnosis of CoQ10 nephropathy and timely initiation of CoQ10 supplementation. Although the majority of patients harbored *COQ8B* variants, our cohort included recurrent and less frequently reported variants and incorporated follow-up data from multiple referral centers across Japan, providing insights into real-world clinical practice. The predominance of a recurrent *COQ8B* variant in this cohort likely reflects population-specific genetic architecture in the Japanese population.

The mechanisms underlying the renoprotective effects of CoQ10 supplementation are multifactorial. CoQ10 plays a central role as an electron carrier in the mitochondrial respiratory chain, and its deficiency disrupts oxidative phosphorylation, leading to impaired ATP production and increased generation of reactive oxygen species [6, 7]. These bioenergetic deficits contribute to podocyte injury, cytoskeletal disorganization, and glomerulosclerosis [9]. Supplementation is thought to restore mitochondrial function, reduce oxidative stress, and preserve podocyte architecture, thereby lowering proteinuria. These considerations support the concept of a time-sensitive therapeutic window for CoQ10 supplementation, in which earlier initiation may maximize the potential reversibility of mitochondrial dysfunction-related podocyte injury before irreversible structural damage occurs.

Our findings should also be interpreted in the context of previously reported international cohorts of CoQ10 nephropathy. In a large multicenter study by Drovandi et al. [19], which included 60 patients with COQ8-associated nephropathy, a ≥ 50% reduction in proteinuria was achieved in 59% of patients, and complete remission was observed in 16%. In comparison, a higher proportion of patients in our cohort achieved marked proteinuria reduction. Preservation of kidney function also appeared favorable. The median baseline eGFR in our cohort (80.9 mL/min/1.73 m^2^) was comparable to that reported in treated *COQ8B* patients (91.5 mL/min/1.73 m^2^). In that study, treated patients exhibited a median annual eGFR decline of 7.2 mL/min/1.73 m^2^/year, whereas untreated patients showed a decline of 17.7 mL/min/1.73 m^2^/year. In contrast, the annualized eGFR decline in our cohort was − 1.7 mL/min/1.73 m^2^/year, with no progression to end-stage kidney disease during follow-up. The median interval from disease onset to treatment initiation was comparable between cohorts, suggesting that timing relative to disease onset alone does not explain the observed differences. However, the age at initiation of CoQ10 supplementation was slightly younger in our pediatric cohort (median 8.5 years, IQR 5.0–9.9) than in the international *COQ8B* cohort (median 11 years, IQR 8.1–17.2), which may have contributed to improved short-term renal outcomes. This difference may partly reflect earlier detection of asymptomatic proteinuria through Japan’s nationwide school urinary screening program, enabling earlier clinical diagnosis and treatment initiation. Several additional factors may also have influenced treatment response. First, the median follow-up duration in our study (28.8 months) was substantially shorter than that reported by Drovandi et al. [19], potentially limiting detection of late disease progression. Second, genetic background may have played a role. Approximately 30% of patients in the international cohort carried biallelic truncating variants, whereas most patients in our cohort harbored missense variants, which may allow partial preservation of protein function and greater responsiveness to CoQ10 supplementation. Finally, the small sample size of our study may have resulted in overestimation of treatment effects. Taken together, our findings support the short- to intermediate-term effectiveness of CoQ10 supplementation, but longer follow-up in larger and genetically diverse cohorts is required to confirm long-term renal outcomes. Only a single patient with a *COQ6* variant was included in this cohort. Therefore, no conclusions regarding genotype-specific differences in treatment response can be drawn. Nevertheless, the marked reduction in proteinuria observed in this patient highlights the potential responsiveness of the patient with a *COQ6* variant to coenzyme Q10 supplementation and indicates the need for larger, gene-specific studies. In addition, several patients received concomitant renin–angiotensin system inhibitors, which may have contributed additively to the observed reduction in proteinuria. Because most patients received concomitant renin–angiotensin system inhibitors, the independent effect of CoQ10 supplementation cannot be fully distinguished from that of background antiproteinuric therapy. However, CoQ10 supplementation was initiated specifically in response to the genetic diagnosis, and the observed reductions in proteinuria are consistent with previous reports supporting its disease-specific therapeutic effect. Determining the independent and synergistic effects of these therapies warrants further investigation.

From a clinical and public health perspective, CoQ10 nephropathy is exceptional among hereditary kidney diseases in that it is diagnosable by genetic testing and treatable with an oral, well-tolerated pharmacological agent. Timely genetic testing in patients with steroid-resistant nephrotic syndrome, focal segmental glomerulosclerosis, or otherwise unexplained proteinuria—especially in the context of a relevant family history—should be strongly considered. Our findings provide real-world evidence that may support regulatory approval and insurance coverage of CoQ10 therapy for genetically confirmed cases, potentially improving access to treatment across Japan and internationally.

This study has several limitations inherent to its retrospective design, including the potential for selection bias and incomplete follow-up in some patients. Although the UPCR was measured using standardized methods across centers, the dosing of coenzyme Q10 varied among patients, reflecting real-world clinical practice. Furthermore, because no standardized or insurance-approved coenzyme Q10 formulation is available, different oral preparations were used, which may have resulted in interindividual variability in absorption and bioavailability. Serum coenzyme Q10 concentrations were not routinely measured, precluding assessment of the relationship between systemic exposure and treatment response. Treatment adherence was also assessed on the basis of clinical records rather than standardized quantitative measures. In Japan, CoQ10 supplementation is not available as an insurance-approved prescription medication and must be obtained independently by patients. This situation may impose financial and logistical burdens, which could adversely affect treatment adherence and consistency. This structural limitation represents an important challenge in the long-term management of CoQ10 nephropathy and highlights the need for the development and regulatory approval of standardized pharmaceutical-grade CoQ10 formulations to ensure equitable access and optimal treatment adherence. Finally, the relatively small sample size limited statistical power and precluded detailed analyses of factors affecting treatment efficacy, particularly within specific genotype subgroups. Nevertheless, the nationwide scope, standardized data collection template, and relatively long follow-up strengthen the credibility of our findings.

In conclusion, CoQ10 supplementation in patients with genetically confirmed CoQ10 nephropathy is associated with a meaningful reduction in proteinuria, stabilization of kidney function, and minimal adverse effects. These findings indicate the importance of an early genetic diagnosis and prompt initiation of targeted therapy. Future prospective, multicenter studies with larger sample sizes, longer follow-up, and standardized outcome measures are warranted to confirm the long-term efficacy, determine genotype-response relationships, and optimize dosing strategies for this rare but treatable form of hereditary kidney disease.

## Supplementary Information

Below is the link to the electronic supplementary material.

Supplementary file1 (DOCX 834 KB)
