# Supplementary material for: Kidney outcomes of coenzyme Q10 supplementation in patients with genetically confirmed CoQ10 nephropathy in Japan
Source: Clin Exp Nephrol. 2026 Jul 11;30(9):1341–9. doi: 10.1007/s10157-026-02917-7 (PMC13518395; doi:10.1007/s10157-026-02917-7)
Supplement: Supplementary file 1 [file 10157_2026_2917_MOESM1_ESM.docx]

Supplementary materials

**Kidney Outcomes of Coenzyme Q10 Supplementation in Patients with Genetically Confirmed CoQ10 Nephropathy in Japan**

China Nagano^1^, Katsumi Ushijima^2^, Yuko Tezuka^3^, Takayuki Okamoto^4^, Yasuhiro Inaba^5^, Akinori Miyazono^5^, Naoko Takeda^6^, Taro Aoki^7^, Akira Mizutani^8^, Hiroki Miyano^9^, Masaki Yamamoto^10^, Mariko Imai^11^, Takehiko Kawaguchi^11^, Toshiyuki Komiya^12^, Masayuki Ishihara^13^, Yoshiki Nagao^13^, Naoaki Mikami^14^, Shuhei Aoyama^1^, Yuka Kimura^1^, Yuta Inoki^1^, Nana Sakakibara^1^, Tomoko Horinouchi^1^, Tomohiko Yamamura^1^, Shingo Ishimori^1^, Kandai Nozu^1^

1. Department of Pediatrics, Kobe University Graduate School of Medicine, Kobe, Hyogo, Japan

2. Department of Pediatrics, Yokkaichi Municipal Hospital, Yokkaichi, Japan

3. Department of Pediatrics, Ehime Prefectural Imabari Hospital, Imabari, Japan

4. Department of Pediatrics, Hokkaido University Hospital, Sapporo, Japan

5. Department of Pediatrics, Kagoshima University Graduate School of Medical and Dental Sciences, Kagoshima, Japan

6. Department of Medicine, Shiga University of Medical Science, Shiga, Japan

7. Internal Medicine 1, Hamamatsu University School of Medicine, Hamamatsu, Japan

8. Department of Pediatrics, Dokkyo Medical University Saitama Medical Center, Saitama, Japan

9. Department of Pediatrics, Juntendo University Nerima Hospital, Tokyo, Japan

10. Department of Pediatric Nephrology, Seirei Hamamatsu General Hospital, Hamamatsu, Japan

11. Department of Nephrology, National Hospital Organization Chiba Medical Center Chibahigashi National Hospital, Chiba, Japan

12. Department of Nephrology, Japanese Red Cross Otsu Hospital, Otsu, Shiga, Japan

13. Department of Pediatrics, Kochi Medical School, Kochi University, Kochi, Japan

14. Department of Nephrology and Rheumatology, Tokyo Metropolitan Children’s Medical Center, Tokyo, Japan

**Corresponding author**

China Nagano, MD, PhD

Department of Pediatrics, Kobe University Graduate School of Medicine,

7-5-1 Kusunoki-cho, Chuo-ku, Kobe, Hyogo 650-0017, Japan

Tel.: +81-78-382-6090, fax: +81-78-382-6099, e-mail: china@med.kobe-u.ac.jp

Contents

Supplementary Figure S1. Long-term trajectories of proteinuria (UPCR) after initiation of coenzyme Q10 supplementation.

Supplementary Figure S2. Long-term trajectories of kidney function (eGFR) after initiation of coenzyme Q10 supplementation.

Supplementary Figure S3. Longitudinal eGFR trajectories plotted against age.

Supplementary Table S1. Time from first detection of proteinuria (disease onset) to CoQ10 initiation in the analyzed cohort (n=12).

**Supplementary Figure S1. Long-term trajectories of proteinuria (UPCR) after initiation of coenzyme Q10 supplementation.**

**Supplementary Figure S2. Long-term trajectories of kidney function (eGFR) after initiation of coenzyme Q10 supplementation.**

**Supplementary Figure S3. Longitudinal eGFR trajectories plotted against age.**

**Supplementary Figure Legends**

**Supplementary Figure S1. Long-term trajectories of proteinuria (UPCR) after initiation of coenzyme Q10 supplementation.**

Spaghetti plot showing longitudinal UPCR measurements for each patient from treatment initiation. Each line represents an individual patient, and dots indicate individual measurement time points. The x-axis denotes months from initiation of coenzyme Q10 supplementation, and the y-axis denotes UPCR (g/gCr).

**Supplementary Figure S2. Long-term trajectories of kidney function (eGFR) after initiation of coenzyme Q10 supplementation.**

Spaghetti plot showing longitudinal eGFR measurements for each patient from treatment initiation. Each line represents an individual patient, and dots indicate individual measurement time points. The x-axis denotes months from initiation of coenzyme Q10 supplementation, and the y-axis denotes eGFR (mL/min/1.73 m²).

**Supplementary Figure S3. Longitudinal eGFR trajectories plotted against age.**
eGFR values are plotted against patient age (years), with lines connecting measurements within each patient. Triangles indicate the first eGFR measurement obtained after initiation of CoQ10 supplementation, and crosses indicate measurements obtained before treatment initiation when available.

Supplementary Table S1. Time from first detection of proteinuria (disease onset) to CoQ10 initiation in the analyzed cohort (n=12).

| **Patient** | **Gene** | **Onset age** | **Age at start of CoQ10 supplementation (yr)** | **Time from disease onset to CoQ10 start (mo)** | **Baseline UPCR (g/gCr)** | **Baseline eGFR (mL/min/1.73m²)** | **Group** |
| --- | --- | --- | --- | --- | --- | --- | --- |
| Neph36 | *COQ8B* | 7y | 10.0 | 36.6 | 2.08 | 85.80 | Pediatric |
| Neph56 | *COQ8B* | 3y | 4.6 | 18.8 | 1.92 | 97.64 | Pediatric |
| Neph97 | *COQ6* | 8m | 1.1 | 4.7 | 50.40 | 125.00 | Pediatric |
| Neph160 | *COQ8B* | 3y | 5.4 | 28.2 | 2.37 | 67.58 | Pediatric |
| Neph364 | *COQ8B* | 21y | 28.4 | 88.4 | 7.94 | 71.00 | Adult |
| Neph501 | *COQ8B* | 6y | 9.8 | 45.5 | 1.30 | 75.79 | Pediatric |
| Neph652 | *COQ8B* | 6y | 7.4 | 16.5 | 1.08 | 92.00 | Pediatric |
| Neph653 | *COQ8B* | 9y | 9.5 | 5.9 | 0.49 | 95.00 | Pediatric |
| Neph814 | *COQ8B* | 9y | 29.2 | 242.7 | 1.79 | 51.20 | Adult |
| Neph859 | *COQ8B* | 34y | 43.5 | 113.9 | 1.09 | 32.30 | Adult |
| Neph868 | *COQ8B* | 7y | 17.6 | 127.0 | 1.43 | 75.90 | Pediatric |
| Neph960 | *COQ8B* | 3y | 3.6 | 7.6 | 6.45 | 108.30 | Pediatric |
